# Amazon Fruits as Healthy Ingredients in Muscle Food Products: A Review

**DOI:** 10.3390/foods13132110

**Published:** 2024-07-02

**Authors:** Juan D. Rios-Mera, Hubert Arteaga, Roger Ruiz, Erick Saldaña, Fernando Tello

**Affiliations:** 1 Instituto de Investigación de Ciencia y Tecnología de Alimentos (ICTA), Universidad Nacional de Jaén, Jaén 06800, Peru; juan.rios@unj.edu.pe (J.D.R.-M.); hubert.arteaga@unj.edu.pe (H.A.); 2 Departamento de Ingeniería de Alimentos, Facultad de Industrias Alimentarias, Universidad Nacional de la Amazonía Peruana, Iquitos 16002, Peru; roger.ruiz@unapiquitos.edu.pe; 3 Sensory Analysis and Consumer Study Group, Escuela Profesional de Ingeniería Agroindustrial, Universidad Nacional de Moquegua, Moquegua 18001, Peru; esaldanav@unam.edu.pe

**Keywords:** Amazon rainforest, fat replacers, fish products, meat products, natural antioxidants

## Abstract

When looking for new ingredients to process red meat, poultry, and fish products, it is essential to consider using vegetable resources that can replace traditional ingredients such as animal fat and synthetic antioxidants that may harm health. The Amazon, home to hundreds of edible fruit species, can be a viable alternative for new ingredients in processing muscle food products. These fruits have gained interest for their use as natural antioxidants, fat replacers, colorants, and extenders. Some of the fruits that have been tested include açai, guarana, annatto, cocoa bean shell, sacha inchi oil, and peach palm. Studies have shown that these fruits can be used as dehydrated products or as liquid or powder extracts in doses between 250 and 500 mg/kg as antioxidants. Fat replacers can be added directly as flour or used to prepare emulsion gels, reducing up to 50% of animal fat without any detrimental effects. However, oxidation problems of the gels suggest that further investigation is needed by incorporating adequate antioxidant levels. In low doses, Amazon fruit byproducts such as colorants and extenders have been shown to have positive technological and sensory effects on muscle food products. While evidence suggests that these fruits have beneficial health effects, their in vitro and in vivo nutritional effects should be evaluated in muscle food products containing these fruits. This evaluation needs to be intended to identify safe doses, delay the formation of key oxidation compounds that directly affect health, and investigate other factors related to health.

## 1. Introduction

The Amazon rainforest occupies nine countries in South America (Bolivia, Brazil, Colombia, Ecuador, Guyana, French Guiana, Peru, Suriname, and Venezuela) with an area of 7 million square kilometers, of which there are an estimated 163 to 250 species of edible fruit plants [1]. Amazon fruits have been gaining scientific interest due to the phytochemicals present in their composition, which have given them the following properties: antioxidant, antimicrobial, antimutagenic, antigenotoxic, analgesic, immunomodulatory, anticancer, bronchodilator, antiproliferative, anti-inflammatory, hypercholesterolemic, leishmanicidal, induction of apoptosis, protective action against diabetes, gastroprotective activity, and antidepressant [2]. For these reasons, the industrialization of Amazon fruits has been encouraged by the cosmetic, pharmaceutical, and food industries [3,4].

Among the food products most questioned due to their potentially harmful effects on health and environmental reasons are meat products; even so, they continue to be foods of high mass consumption. It is well known that these products contain saturated fats and cholesterol, which warns against excessive consumption, since in any of the stages from handling to consumption, oxidation compounds can form with direct health implications. However, oxidation compounds are also attributed to proteins and polyunsaturated fatty acids that are predominant in fish products [5]. Antioxidants of synthetic origin are generally added to delay the formation of oxidation compounds, but these are also questioned due to their potentially harmful effects [6]. In this scenario, the problem of muscle food products (meat and fish products) is doubled.

Muscle food products are complex processed foods manufactured with several ingredients, including meat, lipids, water, salt, and additives, and undergo various unit operations during processing. Removing animal fat or synthetic antioxidants from these products can have technological, bromatological, and sensory effects. Therefore, it is important to define appropriate replacement levels to ensure that the reformulated products still have acceptable characteristics.

This review aims to provide evidence of some fruits that have been studied for their antioxidant properties and their use as fat substitutes, colorants, and extenders in burgers, meatballs, and sausages. We retrieved the articles from the Scopus, Web of Science, Scielo, and EBSCO databases without considering the year of publication. The keywords used were “Amazon fruit AND meat product”, “Amazon fruit AND fish product”, and “Amazon fruit AND muscle food”. The review includes information on the methods used to obtain the ingredients from the fruits, their main characteristics, and their effects on muscle food products. This information can serve as a guide for future studies or industrial purposes. The review also suggests knowledge gaps to be addressed in research related to reforming muscle food products containing Amazon fruits. To this, some studies are cited that report key oxidation compounds harmful to health, and evidence of the positive effects on the health of Amazon fruits is presented, so the characterization of muscle food products must be comprehensive, i.e., considering technological, bromatological, sensory, antioxidant, and in vitro and in vivo nutritional effects.

## 2. Health Issues Related to Muscle Food Products: Lipid Profile and Use of Synthetic Antioxidants

Muscle food products are obtained from edible animals’ skeletal muscles and related tissues and are classified into red meat, poultry, fish, and processed products such as burgers, ham, sausages, meatballs, and surimi, among others [5]. Both processed and unprocessed meats are rich in macro- and micronutrients, including proteins that can exceed the 30% range containing essential amino acids, essential fatty acids (linoleic, linolenic, eicosapentaenoic, and docosahexaenoic acids), vitamins A, D, E, K, B6, B12, biotin, niacin, pantothenic acid, riboflavin, and thiamin, and minerals such as phosphorus, potassium, magnesium, copper, zinc, selenium, and heme iron [7,8,9]. However, the consumption of meat and processed meats, primarily red meat, is controversial and has been linked to colon, stomach, pancreas, and prostate cancer [10].

One of the factors negatively associated with meat and processed meat is the lipid composition, which includes high proportions of saturated fats and cholesterol. However, the reactions that occur in the lipids leading to their oxidation during the handling, processing, storage, cooking, and digestion of muscle food products must be considered. In this context, even polyunsaturated fatty acids (PUFAs) consumed in potentially beneficial amounts may be subject to lipid oxidation. By knowing the lipid oxidation compounds associated with diseases, strategies can be chosen to counteract their formation.

According to Sottero et al. [11], some key oxidation and disease-associated compounds include oxysterols generated by autoxidation and by enzymatic and non-enzymatic reactions of cholesterol, and some aldehydes such as 4-hydroxynonenal from the non-enzymatic oxidation of *n*-6 PUFAs, and 4-hydroxyhexenal produced by the non-enzymatic oxidation of eicosapentaenoic and docosahexaenoic acids. Among oxysterols, Broncano et al. [12] concluded that regardless of the cooking method (grilled, fried, microwave, and roasted), 7α-hydroxycholesterol and 7β-hydroxycholesterol were formed as the main cholesterol oxidation compounds in *Latissimus dorsi* muscle of Iberian pigs. The review by Wang et al. [5] summarizes other compounds that include malondialdehyde, which is often identified in lipid oxidation studies of meat products. These authors also show that oxidation compounds have harmful effects on health and are not exclusive to lipids but also proteins. The contribution of advanced glycation end products and trimethylamine N-oxide must also be considered. From this perspective, the combination of these oxidation sources is crucial for research. For example, Angeli et al. [13] suggested that the interaction of hemoglobin with linoleic acid hydroperoxides could cause DNA damage in human colon adenocarcinoma cell lines and may be associated with the initial stages of cancer.

The food industry typically adds synthetic antioxidants to prevent the oxidation of muscle food products. These antioxidants have maximum usage levels established by health agencies such as the Food and Agriculture Organization through the Codex Alimentarius standards. Some synthetic antioxidants used in muscle food products are butylated hydroxy anisole (BHA), butylated hydroxytoluene (BHT), propyl gallate, and tetra butyl hydroquinone (TBHQ). According to the Codex Alimentarius [14], the maximum level of use of these antioxidants does not exceed 200 mg/kg. All of them are applicable for meat and fish products, except TBHQ, which is only applicable for meat products and fish oil (Table 1).

According to Kumari et al. [6], these additives can cause hypersensitivity, asthma, and cancer. Xu et al. [15] reinforce this idea in their review of the effects of synthetic antioxidants in animal models, where they conclude that BHT promotes tumorigenesis and BHA and TBHQ are related to carcinogenicity processes. However, these authors indicate that there must be more studies in humans to precisely understand the mechanism of action of synthetic antioxidants, which could be different from those observed in animals. However, beyond this gap in knowledge, the risks of high exposure to synthetic additives are latent, which has led researchers to explore the possibility of using natural sources to delay the oxidation of muscle food products.

## 3. Potential Health Benefits of Amazon Fruits

Reports suggest that Amazon fruits have the potential to prevent the formation of oxidation compounds harmful to health. For example, cocoa (*Theobroma cacao* L.) bean shell extracts rich in (−)-epicatechin and tannins, in concentrations between 10 µg/mL and 50 µg/mL, can prevent the oxysterol-induced inflammation [16]. Açai (*Euterpe oleracea* Mart.) reduced 4-hydroxynonenal levels in flies fed a diet containing 2% açai [17]. Likewise, phenolic compounds, of which Amazon fruits stand out, can inhibit advanced glycation end products and trimethylamine N-oxide [18,19]. In addition to phenolic compounds, Amazon fruits stand out for containing unsaturated fatty acids, carotenoids, phytosterols, tocopherols, minerals, essential amino acids, and fibers [2,3,20,21]. From a technological standpoint, these beneficial components are attractive for use in processing muscle food products, as they can replace synthetic antioxidants and animal fat. Some Amazon fruits may have even greater benefits as their health advantages have been studied in vitro, in animal models, and humans.

Açai (*Euterpe oleracea* Mart.) is a fruit from the Amazon region that has been extensively studied and has shown satisfactory results. In a study by Barbosa et al. [22], the effect of consuming 200 g/day of açai pulp for four weeks was investigated in healthy women. The total phenolic compounds of açai pulp were 131 mg gallic acid equivalent/100 g. The results showed increased catalase activity, total antioxidant capacity, and total serum sulfhydryl groups while reducing the production of reactive oxygen species and serum concentration of protein carbonyl. Another study by Udani et al. [23] involved 10 overweight adults who consumed 100 g of açai pulp twice daily for one month. The açai pulp contained 3.5 mg/mL total phenolics measured as gallic acid equivalents and 0.77 mg/mL total anthocyanins measured as cyanidin-3-glucoside equivalents. The results showed reduced total cholesterol and low-density lipoprotein (LDL) cholesterol. A study by Romualdo et al. [24] found that a low-fat diet containing 5% spray-dried açai pulp can attenuate the initiation phase of colon cancer in mice. The compounds of interest were cyanidin 3-glucoside, cyanidin 3-rutinoside, lutein, α-carotene, and β-carotene.

Rambutan (*Nephelium lappaceum* L.) is a fruit that has potential health benefits, mainly due to its peel. In a study by Muhtadi et al. [25], the antidiabetic and antihypercholesterolemia activity of rambutan peel extracts in diabetic rats was studied. The results showed that the highest percentage reduction in blood glucose and cholesterol levels were 61.76 ± 4.26% and 60.75 ± 8.26%, respectively, at 500 mg/kg body weight doses. However, the compounds involved and mechanisms of action were limitations in that study. In this regard, some authors attributed the beneficial effects of rambutan to geraniin. Chung et al. [26] extracted geraniin from rambutan peel and evaluated supplementation for four weeks of 10 and 50 mg/kg body weight in rats receiving a 60% high-fat diet. The results showed a reduction in obesity, visceral fat deposition, triglyceride accretion, and an improvement in insulin resistance. Likewise, Palanisamy et al. [27] reported that geraniin (20 µg/mL) extracted from rambutan peel presented in vitro hypoglycemic activity, aldol reductase inhibition activity and can prevent the formation of advanced glycation end products. However, despite the benefits shown in those studies, the mechanism of action and metabolism of rambutan is poorly understood in humans [28].

Camu camu (*Myrciaria dubia* McVaugh) extracts, rich in phenolic compounds and vitamin C, were administered to diabetic rats for 30 days. The rats received doses of 1 and 3 g/kg/day of camu camu extracts. Each dose contained 2.19 mg and 6.57 mg of phenolic compounds, respectively, and 4.72 and 14.17 mg of vitamin C, respectively, per day. As a result, both doses increased the plasma antioxidant capacity while lipid peroxidation, triacylglycerol, and cholesterol decreased [29]. In humans, camu camu was shown to reduce oxidative stress and biomarkers of inflammation in smokers who were administered daily doses of 70 mL of camu camu juice containing 1050 mg of vitamin C for 7 days [30]. Daily consumption of cupuassu (*Theobroma grandiflorum* Willd. Ex Spreng) liquor for 40 days in diabetic rats (doses of 3.6 and 7.2 g/kg body weight) reduced lipid peroxidation and increased antioxidant capacity. These results were superior to those observed in the cocoa liquor, which had a higher content of polyphenols, caffeine, and saturated fatty acids than the cupuassu liquor [31]. On the other hand, Carvalho et al. [32] reported that red-peel peach palm fruit increased HDL cholesterol and lowered the body mass index in lactating rats. However, the amount of peach palm used in the diet and the compounds of interest are not detailed.

The fruit of the buriti palm (*Mauritia flexuosa* L.) is said to be effective for its antioxidant, prebiotic, and anti-inflammatory properties. Researchers [33] have found that buriti, açai, and cupuassu fruits have antioxidant capacity associated with phenolic compounds. Additionally, açai and buriti promote prebiotic effects by increasing propionate production. All three fruits have shown different protective effects against intestinal inflammation induced by trinitrobenzenesulphonic acid in rats fed diets containing 10 g of buriti, açai, or cupuassu pulp, acting as antioxidant products, increasing mucin and short-chain fatty acids, and decreasing the release of proinflammatory mediators [33]. However, there is no evidence of the effect of cupuassu, peach palm, and buriti in clinical studies in humans.

Lipids, such as sacha inchi oil (*Plukenetia volubilis* L.), were also tested, and they were found to be rich in omega-3 fatty acids. A pilot study conducted by Garmendia et al. [34] found that daily doses of 5 and 10 mL of sacha inchi oil for four months reduced total cholesterol and non-esterified fatty acids in patients with hypercholesterolemia. Additionally, sacha inchi oil increased high-density lipoprotein (HDL) cholesterol in both doses.

Seeds also have biological activities. Guarana (*Paullinia cupana* Kunth) was tested in healthy overweight subjects. Twelve participants consumed 3 g of guarana seed powder (catechin 20 mg/g, epicatechin 30 mg/g) in 300 mL of water before breakfast every morning for 15 days. Guarana increased plasma oxygen radical absorbance capacity (ORAC) and reduced LDL oxidation and hydrogen peroxide-induced DNA damage in lymphocytes, concluding that guarana reduces oxidative stress [35]. Annatto (*Bixa orellana* L.) is another fruit available in the Amazon, and it is widely used as a colorant in the food industry. It has been reviewed that annatto presents biological activities such as antioxidant, free radical scavenging, anti-inflammatory, anticarcinogenic, antibacterial, antifungal, and enhanced gastrointestinal motility, attributed to the carotenoids, apocarotenoids, terpenes, terpenoids, sterols, and aliphatic compounds [36].

The biological activities of Amazon fruits suggest that they could lessen the harmful effects of consuming muscle food products, such as lipid oxidation and processes related to cancer. However, the evaluation of the effects under the conditions of food consumption is a necessity. An example is the study reported by Martínez et al. [37], who added grape seed extracts to turkey and pork emulsions. They found that 0.5% of the extract in the emulsions prevented lipid oxidation before and after simulated gastric digestion. However, the antinutritional effects of fruit components must be considered. In this regard, Santos et al. [38] found that peach palm flour had cytotoxic potential in L929 cells and inhibited protein digestion in vitro in a yogurt model (containing 25% *w*/*w* peach palm flour), which was presumably attributed to the phenolic extract, which contained 2.11 mg gallic acid equivalent/g of dry matter. The authors suggested that the evaluation of the antioxidant capacity is insufficient to demonstrate the functional appeal of reformulated foods, but rather, the adequate levels of the fruit must be supported by toxicological assays. In this line, there is a research opportunity to explore how Amazon fruits can improve muscle food products, considering nutritional studies in vitro and in vivo.

## 4. Amazon Fruit Ingredients in Muscle Food Products

### 4.1. Antioxidants

It is widely known that lipid and protein oxidation are the main causes of deterioration of meat products. The mechanisms of both these processes were recently reviewed by Hadidi et al. [39]. Fish products are particularly vulnerable to lipid oxidation due to their high content of PUFAs, which can cause off-flavors and loss of nutritional value [40]. To solve this problem, researchers have turned to natural plant-based antioxidants. These can be obtained from various parts of plants, such as seeds, peels, leaves, husks, stems, and roots, in the form of powders, juices, and extracts [39,41].

The antioxidant activity of plants can be attributed mainly to their phenolic compounds. These compounds can help to delay primary and secondary lipid oxidation, inhibit lipoxygenase activity, improve color stability, and minimize the degradation of myofibrillar proteins and sulfhydryl groups [42]. Additionally, they have been shown to slow bacterial growth in muscle food products [42,43].

Table 2 lists studies that used native plants found in the Amazon and their effects on meat products. These plants include açai, guarana, and annatto in the form of powders or liquid extracts. As physiological benefits of the antioxidant extracts obtained from these fruits or pigments (in the case of annatto), it has been reported that they can reduce the production of reactive oxygen species (ROS) and reactive nitrogen species (RNS) [35,36,44], of which the mechanism of action of açai has been explained in greater detail by Laurindo et al. [44], where in addition to the reduction of ROS and RNS, açai exerts direct radical scavenging activities, causes a decrease in oxidative leukocytes activation, increases the action of the antioxidants catalase, glutathione peroxidase-1, glutathione peroxidase-4, and superoxide dismutase 1, and increases the nuclear transcription factor-erythroid 2-related factor 2-extracellular signal-regulated kinases-mediated antioxidant pathway, thus acting in processes of brain dysfunctions, aging, cardiac function, and cancer development.

For muscle food relevance, the preparation methods of açai, guarana, or annatto range from simple grinding and sieving to more complex processes like dehydration, extraction with water, ethanol, or a mixture of these, or ultrasound-assisted extraction. The preparation involves agitation, centrifugation, filtration, evaporation, storage for periods of time, and freeze-drying. Some studies also used commercial products such as açai extract powder and norbixin [45,46] (Table 2). The use of commercial products can help to ensure the reproducibility of the effect of the antioxidants evaluated. This is because the composition of the polyphenols and their antioxidant and antimicrobial activities are affected by the geographical area where the plants are collected, making the application of natural antioxidants difficult in the meat industry [42].

The studies evaluated the characteristics of antioxidants such as total phenolic compounds or carotenoids (bixin, norbixin) and measured antioxidant activities using DPPH (2,2-diphenyl-1-picryl-hydrazyl), ABTS (2,2′-azino-bis-3-ethylbenzthiazoline-6-sulphonic acid) or FRAP (ferric reducing antioxidant power). It is difficult to directly compare the total phenolic compounds and antioxidant activities of different Amazon fruits due to the various forms of antioxidants, analysis conditions, and mechanisms of action. Moreover, numerical differences between antioxidants for the phenolic compounds and the same method of antioxidant activity may not necessarily result in an advantage when evaluated in meat products. This was observed by Packer et al. [47], in which guava pomace extract effectively reduced lipid oxidation of chicken meat stored in aerobic packages under refrigeration despite the lower concentration of phenolic compounds and antioxidant activity compared to beetroot stem extract.

The positive effects of antioxidants in meat products are typically found in concentrations ranging from 250 to 500 mg/kg or from 0.025 to 0.05% in percentage terms. However, Şen and Kılıç [48] used up to 9% concentrations of açai extract that was embedded in whey protein isolate-based edible coatings for coating meatballs. The studies cited in Table 1 showed that açai reduced lipid oxidation and did not change the color of pork burgers (250 mg/kg) [49], reduced the oxidation of PUFAs and changes in volatile compounds [45]; also, açai retarded microbial growth in meatballs under refrigeration and freezing [48]. Guarana did not affect the color of pork burgers at low doses (250 mg/kg), being more effective than the synthetic antioxidant (BHT) in delaying lipid and protein oxidation [50]. It also delayed discoloration and did not affect the sensory characteristics of lamb burgers [51]. Annatto (0.05%) reduced the formation of thiobarbituric acid reactive substances and cholesterol oxidation in pork patties. However, the combination with sodium erythorbate was favorable to prevent the degradation of bixin [52]. Similarly, norbixin from annatto prevented the oxidation of sausages [46].

Therefore, using antioxidants derived from Amazon fruits can offer technological benefits for producing meat products that are less harmful to health. Still, further studies are needed to explore the effects of these antioxidants on fish products and their in vitro and in vivo nutritional effects, as described in Section 3 of this review. Additionally, other sources of antioxidants are available in the Amazon biome that can be explored. However, to ensure the accuracy of the results and a better understanding of the mechanism of action of natural antioxidants, it is essential to follow the criteria outlined by Estévez [53], which includes careful planning of the experiment and experimental design, selection of appropriate sources of antioxidants, identification of bioactive compounds, study of the molecular mechanism, potential benefits, and safe doses. Regarding this last aspect, beyond the low or no toxicity reviewed by some authors [36,44], it is necessary to study the limits of the use of natural antioxidants added to foods through toxicological tests. A good example is the previously cited work of Santos et al. [38], related to the toxicity and decreased protein digestibility of yogurt due to the phenolic compounds of peach palm. Thus, to define appropriate levels of antioxidants, future studies should follow that line of research.

**Table 2:** Amazon fruits as antioxidants in muscle food products.

Amazon Resource	Ingredient Preparation Method	Characteristics of the Ingredient	Main Findings	Reference
Açai extract powder	- Water addition to the açai pulp at a 1:1 ratio. - Agitation (30 min at 150 rpm) protected from light. - Centrifugation (4500 rpm for 20 min). - Filtration of supernatant and freeze-drying.	- Total phenolic compounds: 612.54 ± 17.64 mg of GAE/g of extract. - Antioxidant activity (DPPH—2,2-diphenyl-1-picryl-hydrazyl): 47.57 ± 0.83 μmol Trolox equivalents/g of açai extract.	250 mg/kg of açai extract did not affect the color of pork burgers. Furthermore, it was comparable to sodium erythorbate (500 mg/kg) in reducing lipid oxidation.	[49]
Açai extract powder	Commercial açai extract powder	- Total phenolic compounds: 31.36 ± 1.220 mg gallic acid/g of sample. - ABTS—2,2′-azino-bis-3-ethylbenzthiazoline-6-sulphonic acid: 50.54 ± 0.296 mg ascorbic acid/g of sample. - FRAP—ferric reducing antioxidant power: 38.05 ± 1.268 mg ascorbic acid/g of sample.	Reduction of the oxidation of polyunsaturated fatty acids and lower changes in volatile compounds of reduced-fat beef burgers.	[45]
Açai extract	- 10 g of commercial açai powder mixed with 100 mL ethanol (100%). - Stored at room temperature and dark condition for 24 h. - Filtration of supernatants and evaporation for 10 min.	- Total phenolic compounds: 36.8 mg gallic acid equivalents/g of sample. - DPPH: 277.83 mg/mL. - FRAP: 1305 μmol/L Fe^+2^	Açai extract in whey protein isolate-based edible coatings suppressed microbial growth (total viable aerobic count and coliforms) in meatballs stored at 4 °C and −18 °C.	[48]
Guarana seeds	- Dispersion of 1 g seed in 10 mL hydroethanolic solvent (40:60, water: ethanol). - Extraction in ultrasonic bath for 45 min (room temperature and darkness). - Magnetic stirring at 80 °C for 30 min. - Filtration, evaporation, and freeze-drying.	- Total phenolic compounds: 258 mg gallic acid equivalents/g of extract. - Compounds: tyrosols, phenolic acids, anthocyanins, alkylphenols, flavonols, flavones, stilbenes, and lignans. - DPPH: 0.3 g/L. - ABTS: ~2072 μmol Trolox/g of extract.	Guarana seed extract did not affect the color of pork burgers at the lower dose (250 mg/kg). The antioxidant power in lipids and proteins was more effective in the extracts than the synthetic antioxidant (BHT). However, the natural extract was not effective as an antimicrobial.	[50]
Guarana seeds	- Drying in a force air-circulating oven at 40 °C for 72 h. - Grinding and sieving (48 mesh). - Other processes are the same of Pateiro et al. [50].	Not determined.	250 mg/kg guarana extract delayed discoloration, retarded lipid and protein oxidation and did not affect the sensory characteristics of lamb burgers.	[51]
Annatto seeds	Grinding and sieving (100 mesh).	Bixin content: 14 ± 2 mg/g.	Annatto (0.05%) was effective in reducing the formation of thiobarbituric acid reactive substances and cholesterol oxidation in pork patties after thermal treatment. Sodium erythorbate combined with annatto protected bixin from degradation.	[52]
Norbixin from annatto	Commercial norbixin	Norbixin represented 10% of the annatto composition.	Norbixin was efficient in preventing lipid oxidation of sausages.	[46]

### 4.2. Fat Replacers

Animal fat is commonly used in the production of meat products due to its ability to form emulsions with proteins, improve yield, and enhance sensory characteristics such as flavor, tenderness, and juiciness [54]. However, some meat products can contain up to 30% fat [55], which can develop potentially harmful compounds. To address this issue, botanical sources such as vegetable flours, edible mushrooms, and healthy oils can be used as alternatives to animal fat. These sources can be added directly or contained in microcapsules, microparticles, emulsions, hydrogels, oleogels, and bigels [54,56]. Studies have been conducted using native sources or those found in the Amazon biome to replace animal fat in meat products. Table 3 details that these studies used gels, oils, and flours.

Botella-Martinez et al. [57] used cocoa bean shell flour to create gelled emulsions by mixing, emulsifying, and storing for gel formation. The gels were then used to replace 50% and 100% animal fat in beef burgers. Cocoa bean shell flour was chosen due to its high fiber content and compounds of interest, such as catechin, epicatechin, theobromine, and caffeine. The study found that the burgers were sensorially acceptable at 50% fat replacement and had an increase in cooking yield, linolenic and linolenic fatty acids, and polyunsaturated/saturated fatty acid ratios. Additionally, the fat content, atherogenicity, and thrombogenicity index were reduced. However, lipid oxidation increased due to the gelled emulsion.

Similarly, Hanula et al. [58] added encapsulated açai oil to hydrogel emulsions to replace animal fat in burgers. The process involved agitation, emulsification, and freeze-drying. Similar to Botella-Martinez et al. [57], the emulsions improved the lipid profile and cooking yield, with no sensory differences between the control at 50% fat reduction. However, lipid oxidation tended to increase in the reformulated products. Wongpattananukul et al. [59] also observed a similar effect when adding sacha inchi oil (0.5–1.5 g/100 g of ground chicken) to chicken sausages. The study found an improvement in the lipid profile and technological parameters without effects on sensory acceptability, but there were increases in lipid and protein oxidation. These studies suggest that incorporating antioxidants as a study variable may help counteract lipid and protein oxidation. In this regard, Badar et al. [60] suggest hydrogels have an advantage over oleogels in replacing animal fat in meat products because the former allows the use of hydrophilic and lipophilic antioxidants.

Guzmán et al. [61] used peach palm pulp and peel flours to replace up to 50% of animal fat in beef-based burgers. The study found reduced texture parameters, fat, cooking losses, and diameter reduction. Interestingly, a reduction in lipid oxidation was observed in burgers with peach palm flour, demonstrating that peach palm fruit can act as a fat replacer and as an antioxidant source. It is important to note that the processes used to obtain the peach palm flours are simple, such as cooking, dehydration, and grinding, which can be economically viable for replacing animal fat in meat products.

The flours used as fat substitutes are known for their fiber content, which has the property of retaining water, mainly when the fiber is soluble [62]. However, in the studies cited in Table 3, the type of fiber of the ingredient used as a fat replacer was not quantified or identified, so it is suggested that this aspect be evaluated in future studies.

**Table 3:** Amazon fruits as fat replacers in muscle food products.

Amazon Resource	Ingredient Preparation Method	Characteristics of the Ingredient	Main Findings	Reference
Gelled emulsion containing cocoa bean shell flour	- Mix of gelling agent (5%) with water (40%) at 12,000 rpm for 2 min. - Cocoa bean shell flour (15%) added and mixed in a food processor at 1300 rpm for 2 min. - Walnut oil (40%) gradually added. - Emulsification at 5000 rpm for 5 min. - Stored at 2 °C for 5 h to gel formation.	- Not determined in gelled emulsion. - Cocoa bean shell flour had the following characteristics: total dietary fiber of 61.18 g/100 g; protein content of 17.13 g/100 g; catechin and epicatechin content of 4.56 and 6.33 mg/g, respectively; theobromine and caffeine content of 12.27 and 6.13 mg/g, respectively.	At two levels of animal fat replacement in beef burgers (50 and 100%), there was an increase in moisture and ash, a reduction in fat and proteins, an increase in linolenic and linolenic fatty acids, and in the polyunsaturated/saturated fatty acid ratio, reduction of the atherogenicity index and thrombogenicity index, and increased cooking yield. At 50% fat replacement, the samples were sensorially acceptable. However, at both levels of fat replacement, there was an increase in lipid oxidation.	[57]
Hydrogel emulsion containing encapsulated açai oil	- 2 g sodium alginate and 0.86 g of konjac flour in 100 mL water at 60 °C with stirring. - Emulsion (2:1 oil: water) homogenized in the biocomposite obtained. - Addition of defatted flaxseed flour (5 g flaxseed flour/95 g hydrogel emulsion). - Freeze-drying of the hydrogel with encapsulated açai oil (31%).	Not determined.	Higher content of polyunsaturated fatty acids of about 32%, reduction of saturated fatty acids by 22%, lower atherogenicity index, and thrombogenicity index, increase in hypocholesterolemic/hypercholesterolemic ratio, reduction in cooking losses, and no significant differences in sensory attributes were observed between control and 25 and 50% beef fat reduction in burgers. However, lipid oxidation tended to increase.	[58]
Sacha inchi oil	Commercial sacha inchi oil	Not determined.	By replacing chicken fat with sacha inchi oil (0.5–1.5 g/100 g of ground chicken) in chicken sausages, there was a reduction in saturated fat, omega-6/omega-3 ratio, and the atherogenic and thrombogenic index, increase in omega 3 fatty acids; at 0.5 g sacha inchi, there was an improvement of emulsion stability, no effects in cooking losses texture properties, and sensory acceptability. However, lipid and protein oxidation increased at 1.5 g/100 g sacha inchi oil.	[59]
Pulp and peel flour of peach palm	- Cooking of fruits in boiling water for 30 min. - Separation of pulp and peel. - Drying in an oven with circulating air at 55 °C until moisture less than 15%.	Not determined.	At 25 and 50% fat replacement in beef-based burgers, pulp and peel flours decreased hardness, springiness, cohesiveness, chewiness, fat, cooking losses, and diameter reduction. Lower lipid oxidation was obtained with peel flour.	[61]

### 4.3. Colorants and Extenders

Fruit byproducts available in the Amazon were also used as colorants and extenders in muscle food products, as cited in Table 4. The colorant colorific containing annatto in its composition with a bixin content of 173 ± 24 mg/100 g remained stable. The red and yellow colors in raw and cooked chicken patties also decreased lipid oxidation, but bixin was not stable after cooking [63]. In another study, the combination of annatto powder:nitrite at 60%:40% in sausages did not present significant differences with the control treatment (100% nitrite) for microbial counts and sensory properties and also intensified the red color of the product [64]. These studies, complemented with the antioxidant activity of annatto described in Section 4.1, suggest that annatto can improve the oxidative stability and color of meat products, but it is necessary to optimize the formulations to avoid losses of bixin and thus extend shelf life.

**Table 4:** Amazon fruits as colorants and extenders in muscle food products.

Role/Amazon Resource	Ingredient Preparation Method	Characteristics of the Ingredient	Main Findings	Reference
Colorant/colorifico containing annatto seeds	Commercial colorifico	Bixin content: 173 ± 24 mg/100 g	Stable and intense red and yellow color in both raw and grilled chicken patties containing 0.4 g/100 g colorifico; lipid oxidation delayed in grilled patties; vitamin E higher in raw chicken; bixin was not stable after grilling.	[63]
Colorant/annatto powder	Commercial annatto powder	Norbixin content: 1%	Sausages containing 60%:40% annatto powder:nitrite had higher redness and lower yellowness without presenting significant differences with the control sample (100% nitrite) in microbial counts and sensory properties.	[64]
Colorant/oil extract of peach palm fruit residues	- Drying in an oven at 60 °C until constant moisture level. - Grinding and sieving (0.25 mm). - Ultrasonic assisted extraction.	Total carotenoids varying from 2.61 to 28.13 mg/kg extract, containing 9 to 97 mL/kg extract added to the product.	In Frankfurter sausages, there was an increase in lightness, yellowness, chroma, and hue. On the contrary, redness decreased.	[65]
Extender/pulp flour of peach palm	- Peach palm fruit cooked in boiling water for 40 min. - Separation of peach palm pulp. - Freeze-drying of pulp. - Grinding.	Not determined.	3% of peach palm in tilapia sausages decreased instrumental consistency, adhesiveness, cohesiveness, cutting work, and yield, increased hardness, gumminess, springiness, shear force, and sensory texture, flavor, and odor.	[66]
Extender/cocoa shell powder	- Roasting of cacao samples at 135 °C for 15 min. - Cocoa shell was obtained after mechanically separating the nibs. - Grinding and sieving (0.55 mm) of cocoa shell.	15.2% protein, 15.1% lipid, 48.1% total dietary fiber, 14.7% carbohydrate, 1.9% moisture, 7% ash, 1.10 mg/g epicatechin, 1.04 isoquercetin.	1.5 to 3% cocoa shell powder in burgers increased fiber, lipids, and hardness, decreased weight and volume loss during cooking, no effects in sensory traits, and a slight reduction in *Pseudomonas* was observed.	[67]

Peach palm oil extract was studied as a colorant in sausages. Pinzón-Zárate et al. [65] used peach palm fruit residues to extract oil with the assistance of an ultrasound. The addition of oil to the Frankfurt sausages was a function of the carotenoid content in the oil. As a result, lightness, yellowness, chroma, and hue increased while redness decreased. However, sensory analysis was not studied to determine if these changes are acceptable.

Peach palm can also be used as an extender, as reported by Zapata and de la Pava [66], who observed that freeze-dried peach palm pulp at a concentration of 3% in tilapia sausages increased sensory properties, but the texture properties results were variable. The studies cited on the peach palm in muscle food products suggest that the fruit byproducts of this palm tree are multifunctional; that is, they can act as a fat replacer, extender, and colorant and could have antioxidant activity. Another extender reported was cocoa shell powder by Delgado-Ospina et al. [67]. These authors observed that cocoa shell powder at 1.5 to 3% in burgers improved the proximal and technological profile without effects on sensory characteristics. There was also a slight reduction in *Pseudomonas* bacteria.

**Figure 1:**
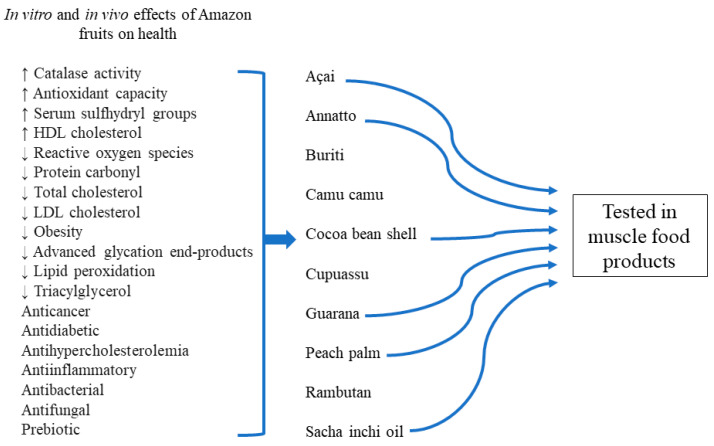
In vitro and in vivo effects of Amazon fruits on health and their use in muscle food products. ↑ means increase and ↓ decrease of the effects of Amazon fruits.

In short, Amazonian fruits improve the characteristics of muscle food products when added as antioxidants, fat replacers, colorants, and extenders. The antimicrobial role is also a feature to consider. In this review, 10 fruits were cited with potential health benefits and can prevent the formation of specific oxidation compounds (detailed in Section 2 and Section 3 of this review), of which only 6 fruits have been incorporated into muscle food products (Figure 1). Therefore, the possibility of continuing to study these and other Amazon fruits is very broad.

## 5. Conclusions

Consumption of muscle food products, including processed meat from red meat, poultry, and fish, can be detrimental to health due to their lipid profile that forms potentially harmful compounds and due to the use of synthetic antioxidants. It is necessary to reformulate these products using botanical sources as ingredients to replace animal fat and synthetic antioxidants, which is the strategy most widely accepted by the scientific community. This review explores the potential use of Amazon fruits as ingredients in muscle food products. As such, they can potentially delay the formation of oxidation compounds. Besides antioxidant properties, Amazon fruits were evaluated as fat replacers, colorants, extenders, and even for their antimicrobial properties. Fruits like annatto or peach palm can have multiple benefits, making them advantageous for replacing more than one ingredient in muscle food products.

**Figure 2:**
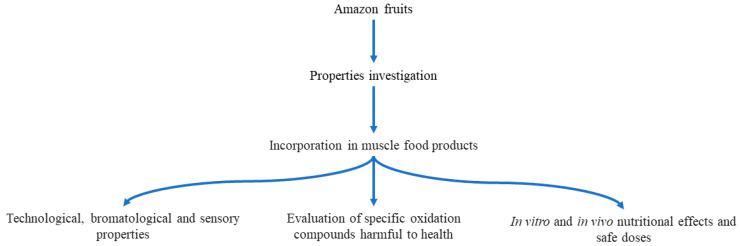
Perspective of studies on Amazon fruits in muscle food products.

Studying the nutritional properties of muscle food products containing byproducts of Amazon fruits could provide functional characteristics to this type of products and the denomination of “healthy foods”. However, the challenge is great, and we suggest considering the aspects shown in Figure 2. First, the fruits must be evaluated regarding their phytochemical, antioxidant, antimicrobial, physicochemical, technological, and bromatological properties. Second, choose the most appropriate function of the fruits according to their characteristics, such as antioxidants, fat replacers, colorants, extenders, or antimicrobials. The combination of these functions is a good option to consider. Third, evaluate the potential to delay oxidation of compounds harmful to health, especially under conditions of product consumption. Finally, nutritional effects in vitro and in vivo should be evaluated to define the functional potential and safe doses. This last aspect is key to taking the next step to face the great gap that exists between the knowledge reported by academia and the industrial application, which is conditioned by regulatory aspects, precisely due to the lack of knowledge of the toxicological effects of new ingredients.

The Amazon rainforest offers a wide variety of fruit species, which presents an opportunity for scientific research to reformulate muscle food products to improve their nutritional profile. Sustainable industrial use of these fruits could provide income and improve the competitiveness of Amazon settlers.

## Figures and Tables

**Table 1:** Maximum level of use of synthetic antioxidants in meat and fish products, according to Codex Alimentarius [14].

Antioxidant	Product	Maximum Level (mg/kg)
BHT	Meat products	100
	Fish and fish products	200
BHA	Meat products	100
	Fish and fish products	200
Propyl gallate	Meat products	200
	Fish and fish products	100
TBHQ	Meat products	100
	Fish oil	200

## Data Availability

No new data were created or analyzed in this study. Data sharing is not applicable to this article.
